# A transcriptomic analysis of *Neurospora crassa* using five major crop residues and the novel role of the sporulation regulator *rca-1* in lignocellulase production

**DOI:** 10.1186/s13068-015-0208-0

**Published:** 2015-02-12

**Authors:** Bang Wang, Pengli Cai, Wenliang Sun, Jingen Li, Chaoguang Tian, Yanhe Ma

**Affiliations:** Key Laboratory of Systems Microbial Biotechnology, Tianjin Institute of Industrial Biotechnology, Chinese Academy of Sciences, Tianjin, 300308 China; University of Chinese Academy of Sciences, Beijing, 100049 China

**Keywords:** *Neurospora crassa*, Crop residues, Transcriptional profiling, Biomass regulon, *rca-1*

## Abstract

**Background:**

Crop residue is an abundant, low-cost plant biomass material available worldwide for use in the microbial production of enzymes, biofuels, and valuable chemicals. However, the diverse chemical composition and complex structure of crop residues are more challenging for efficient degradation by microbes than are homogeneous polysaccharides. In this study, the transcriptional responses of *Neurospora crassa* to various plant straws were analyzed using RNA-Seq, and novel beneficial factors for biomass-induced enzyme production were evaluated.

**Results:**

Comparative transcriptional profiling of *N. crassa* grown on five major crop straws of China (barley, corn, rice, soybean, and wheat straws) revealed a highly overlapping group of 430 genes, the biomass commonly induced core set (BICS). A large proportion of induced carbohydrate-active enzyme (CAZy) genes (82 out of 113) were also conserved across the five plant straws. Excluding 178 genes within the BICS that were also upregulated under no-carbon conditions, the remaining 252 genes were defined as the biomass regulon (BR). Interestingly, 88 genes were only induced by plant biomass and not by three individual polysaccharides (Avicel, xylan, and pectin); these were denoted as the biomass unique set (BUS). Deletion of one BUS gene, the transcriptional regulator *rca-1*, significantly improved lignocellulase production using plant biomass as the sole carbon source, possibly functioning via de-repression of the regulator *clr-2*. Thus, this result suggests that *rca-1* is a potential engineering target for biorefineries, especially for plant biomass direct microbial conversion processes.

**Conclusions:**

Transcriptional profiling revealed a large core response to different sources of plant biomass in *N. crassa*. The sporulation regulator *rca-1* was identified as beneficial for biomass-based enzyme production.

**Electronic supplementary material:**

The online version of this article (doi:10.1186/s13068-015-0208-0) contains supplementary material, which is available to authorized users.

## Background

Renewable plant biomass is a potential low-cost feedstock for the microbial production of lignocellulolytic enzymes, biofuels, or value-added chemicals. This process represents a promising strategy to supply the world’s energy demand and reduce greenhouse gas emissions derived from burning fossil fuels [1,2]. Crop residues are ideal raw materials for bioconversion because they are abundant worldwide, low-cost, compatible with food security, and >50% (*w*/*w*) cellulose/hemicellulose, which can be used for biorefinery [3,4]. However, plants have evolved rigid and complex cell walls to support upright growth, transport nutrients, and protect against microbial invasion. Composed of various polymeric building blocks (typically including cellulose, hemicellulose, pectin, lignin, and structural proteins) [5], the plant cell wall presents a greater challenge to lignocellulolytic enzyme digestion than individual polysaccharides like microcrystalline cellulose (e.g., Avicel), xylan, or pectin, which are commonly used in biorefinery studies. High complexity and heterogeneity in chemical composition and organization makes plant biomass a bioprocessing feedstock with uneven chemical and physical properties [6,7]. Therefore, understanding the commonalities and differences in microbial degradation of diverse plant cell wall types will be useful for developing a case-by-case or universal strategy for plant biomass biorefinery.

Filamentous fungi are currently the main source for commercial carbohydrate hydrolytic enzyme production [1]. Investigation of fungal responses to a wide spectrum of biomass substrates is needed not only to gain more knowledge of plant cell wall degradation and utilization but also to rationally engineer filamentous fungi for biomass-based enzyme, ethanol, and chemical production. This information will be especially helpful in using plant biomass direct microbial conversion (DMC), a recently suggested, promising strategy [7,8] whereby filamentous fungi are used to generate valuable products, such as fatty acids produced by *Neurospora crassa* [9] and ethanol by *Trichoderma reesei* and *Fusarium oxysporum* [10]. Although transcriptome analysis has been performed in different fungal systems digesting various types of plant biomass (such as *Miscanthus giganteus*, barley, oat, canola, alfalfa, wheat straw, and bagasse) [11-16], the comparative analysis of genome-wide profiling on a wide spectrum of biomass substrates in a single fungus species can offer outstanding knowledge on specific and common microbe responses to different crop residues, which will be useful for engineering fungal DMC using various sources of plant biomass.

The filamentous fungus *N. crassa* has the ability to robustly degrade and utilize lignocellulosic materials [12,17]. Its available genome-wide deletion database [18,19] offers great advantages for ongoing studies of the mechanism of digesting polysaccharides (such as Avicel, xylan, and pectin) [12,20-22]. Studies using this model organism have begun to reveal a more thorough perspective on lignocellulolytic enzyme induction, regulation, and production in filamentous fungi [23]. These advances can be rationally applied to *N. crassa* as well as other filamentous fungi to further improve their production of lignocellulolytic enzymes and even target chemicals via DMC [9].

In this study, we report a large-scale comparison of *N. crassa* transcriptome induction by five crop straws (barley, corn, rice, soybean, and wheat straws; BS, CS, RS, SS, and WS, respectively) that are highly abundant in China [24] and around the world [25]. Differential gene expression profiling of *N. crassa* grown on these crop residues versus sucrose showed a large “core” and a small “shell” response to a variety of plant straws. Carbohydrate-active enzymes (CAZys) [26] were significantly induced by all five straws and displayed a highly overlapping gene pool, indicating a conserved CAZy set for plant biomass degradation in *N. crassa*. This CAZy set contained a high proportion (10 of 14) of plant cell wall-induced lytic polysaccharide monooxygenase (LPMO) genes. Finally, screening mutants of transcription factors which were induced only by complex plant cell wall samples yielded a sporulation regulator *rca-1* that was beneficial for lignocellulolytic enzyme production on plant biomass in *N. crassa*.

## Results

### Chemical composition of crop residues

**Figure 1:**
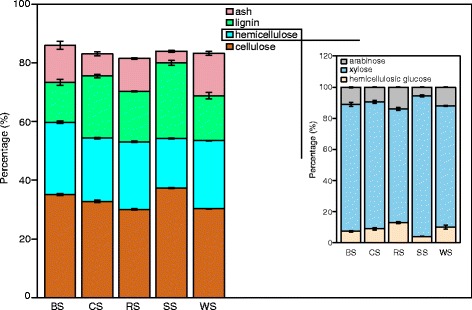
**Chemical composition (left panel) and hemicellulosic component distribution in hemicellulose (right panel) of five crop residues.** BS, barley straw; CS, corn straw; RS, rice straw; SS, soybean straw; WS, wheat straw. Error bars represent the respective standard deviation of three replicates.

The five crop straws contained similar proportions of cellulose (30%–40%; Figure 1 and Additional file 1: Table S1), the major component of agro-residues [3]. Four (BS, CS, RS, and WS) comprised more than 20% hemicelluloses, while soybean straw contained less hemicellulose but the highest lignin content among five crop residues (Figure 1). Moreover, soybean straw had the lowest proportion of arabinose in hemicellulose (5.6%) compared with the other four straws (9.4%–13.8%; Figure 1 and Additional file 1: Table S1). Generally, lignin and ash compositions in the five crop straws were more varied than that of cellulose/hemicellulose (Figure 1). The variety in chemical composition combined with complex organization account for the high heterogeneity in chemical and physical properties of plant biomass.

### Differential expression analysis of biomass-induced profiling data revealed common and unique responses to crop residues

**Figure 2:**
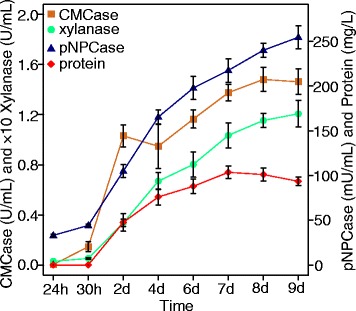
**Growth curve of** ***Neurospora crassa*** **on 2% (** ***w*** **/** ***v*** **) ground corn straw as the sole carbon source.** The cellulose hydrolysis activity (CMCase), exo-glucanase activity (pNPCase), xylanase activity, and total protein in supernatants were determined between 24 h and 9 days. Values represent the means of at least three replicates; error bars show standard deviation.

**Figure 3:**
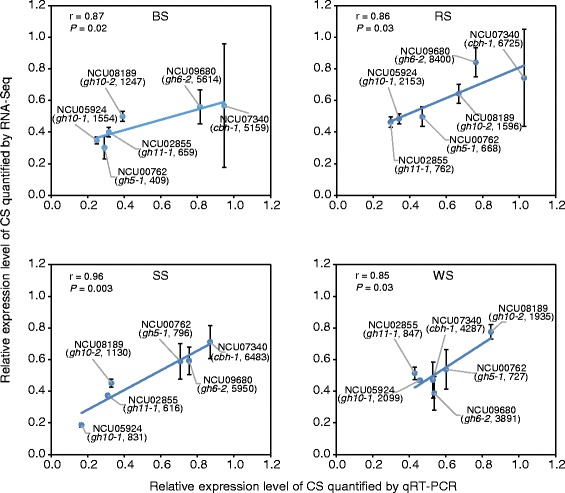
**Correlation comparison between qRT-PCR results and RNA-Seq data.** BS, barley straw; CS, corn straw; RS, rice straw; SS, soybean straw; WS, wheat straw. Relative expression levels of selected CAZy genes were normalized by the expression under CS. Values of the corresponding gene expression levels by RNA-Seq (RPKM) are shown in brackets. Pearson correlation coefficients and *P* values are shown on the top left of each figure. For qRT-PCR (horizontal coordinate), values represent the means of triplicates; error bars show standard deviation.

Cellulase and xylanase activities and secreted proteins of *N. crassa* (FGSC 2489) cultured on ground corn straw were determined over time. Culture supernatant rarely accumulated protein until day 2 (Figure 2), but weak cellulase and xylanase activities could be detected at 30 h (Figure 2). Based on a previous study [12], lignocellulolytic enzymes can be transcriptionally highly induced by plant biomass before 40 h in *N. crassa*. By checking the 30-h cultures grown on each crop residue medium, the mycelia were phenotypically (hyphal length and branching) comparable to the 16-h culture on sucrose (Additional file 2: Figure S1), which were further used for transcriptome profiling (RNA-Seq). RNA-Seq data showed good accordance with the results of quantitative real-time reverse transcription polymerase chain reaction (qRT-PCR) using the Pearson correlation analysis (Figure 3). All selected cellulases (NCU07340, *cbh-1*; NCU09680, *gh6-2*; and NCU00762, *gh5-1*) and hemicellulases (NCU08189, *gh10-2*; NCU05924, *gh10-1*; and NCU02855, *gh11-1*) were highly induced after 30 h of growth on crop residues (Figure 3).

**Figure 4:**
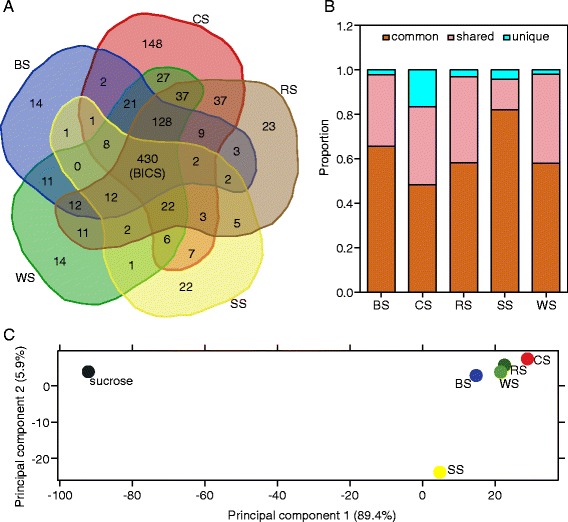
**Overview of** ***Neurospora crassa*** **transcriptome induced by five crop residues.** BICS, biomass commonly induced core set; BS, barley straw; CS, corn straw; RS, rice straw; SS, soybean straw; WS, wheat straw. **(A)** Venn diagram of genes upregulated by each crop straw. **(B)** Proportions of genes upregulated by each crop straw. “Common” represents genes induced by all five straws; “shared” represents genes induced by at least two but not all straws; “unique” represents genes uniquely induced by one crop residue. **(C)** Principal component analysis of transcription profiling data performed by DESeq. The RNA-Seq reads of *N. crassa* grown on sucrose, BS, CS, RS, SS, and WS were used for the principal component plot. Each point on the graph represents one biological sample. Proportions of the principal components 1 and 2 are shown in brackets.

For further analysis, differential gene expression analysis was performed by generalized fold change (GFOLD) [27] and DEGseq [28] using the 16-h sucrose-grown sample as a control (Additional file 3: Table S2; Methods). Totals of 520–890 genes were significantly elevated, with greater than threefold changes (GFOLD ≥ 1.6, *P* value < 1e-4) in the five substrates compared with the sucrose condition (Table 1). Of these genes, 430 genes were commonly induced on all five straws (Figure 4A). Conversely, a small number of genes (76–253 genes) showed decreased expression based on the same criteria (GFOLD ≤ −1.6, *P* value < 1e-4) (Table 1). For those upregulated genes, approximately 60% (300–560 genes) also exhibited threefold upregulation when mycelia pre-grown on sucrose for 16 h were transferred to minimal medium (“Methods”) with no-carbon source for 4 h (Table 1), indicating a starvation response that was synergic with plant cell wall degradation and utilization in *N. crassa* [20]. However, these expression changes under the no-carbon condition were not of the same order of magnitude as those under the lignocellulosic conditions (Additional file 3: Table S2).

**Table 1:** **Differential gene expression profile on five crop residues**

	**BS**	**CS**	**RS**	**SS**	**WS**
Upregulated genes	656	888	738	524	742
“Common” rate^a^	65.5%	48.4%	58.3%	82.1%	58.0%
“No-carbon” rate^b^	60.7%	63.6%	62.6%	57.6%	62.1%
Downregulated genes	76	253	117	141	101
“Common” rate	72.4%	21.7%	47.0%	39.0%	54.5%
“No-carbon” rate	60.5%	78.3%	63.2%	46.1%	63.4%

*BS* barley straw, *CS* corn straw, *RS* rice straw, *SS* soybean straw, *WS* wheat straw.

^a^Genes commonly regulated by all five straws versus sucrose as a sole carbon source; “no-carbon” data were derived from a reference [20].

^b^Genes regulated in the no-carbon condition versus sucrose as a sole carbon source.

Except the 430 biomass commonly induced core set (BICS) genes, many genes showed different induction patterns under the five straws. For example, 148 genes were specifically induced by corn straw, accounting for a greater percentage of all induced genes than in other crop straws (Figure 4B). Most “CS-unique” genes (85 of 148) were also induced by the no-carbon condition (Additional file 4: Table S3, sheet 1), suggesting that corn straw may elicit broader carbon de-repression than other crops. Conversely, soybean straw induced the fewest genes (Table 1), as well as “shared” and “unique” genes (Figure 4A, B). Furthermore, a second large set of shared genes in Figure 4A (128 genes induced in BS, CS, RS, and WS) was not upregulated on soybean straw. This feature was also verified by principle component analysis (PCA) based on transcriptome expression profiling, which showed that soybean straw (a dicot crop) was separated a little from the other four monocot straws (Figure 4C). These observations might reflect the transcriptional responses of *N. crassa* to the chemical composition distinctions displayed in Figure 1.

A detailed functional analysis (FunCat [29]) showed that polysaccharide or carbohydrate metabolism was also enriched in all five biomass “shared” and “unique” sets, which was not the case in the “common” set or starvation condition (no carbon) (Additional file 4: Table S3, sheet 1). Although these 23 polysaccharide/carbohydrate metabolism genes (Additional file 4: Table S3, sheet 1, after removing redundant ones) in the “shared” and “unique” sets induced by each straw were not the same, most (17 out 23) were induced by at least two crop straws. For example, all six CAZy genes (NCU09904, *gh16*; NCU09175, *gh17*; NCU09042, *gh2*; NCU07351, *gh67*; NCU04526, *ce3*; and NCU01059, *gh47*) were upregulated under at least two straw conditions (Additional file 4: Table S3, sheet 1). However, corn straw uniquely induced many genes unrelated to C-compound metabolism but associated with secondary metabolism and detoxification (Additional file 4: Table S3, sheet 1). For example, NCU05780, a glutathione S-transferase gene, was highly induced only on corn straw (RPKM (reads per kilobase per million mapped reads) = 224.6, Additional file 3: Table S2), indicating a reduction reaction was required for detoxifying oxides when *N. crassa* was grown on corn residue. Intriguingly, rice straw induced some cation-metabolism genes and soybean straw induced nucleoside-metabolism genes, reflecting diverse impurities, such as iron contamination [15], in different crop straws. Functional analysis of all downregulated genes in the five straws revealed that protein-folding, structural protein, inorganic element metabolism, and disease-defense genes were mainly enriched in this gene set (Additional file 4: Table S3, sheet 2).

### Comparative analysis of biomass regulon revealed common and specific features with polysaccharides regulons

**Figure 5:**
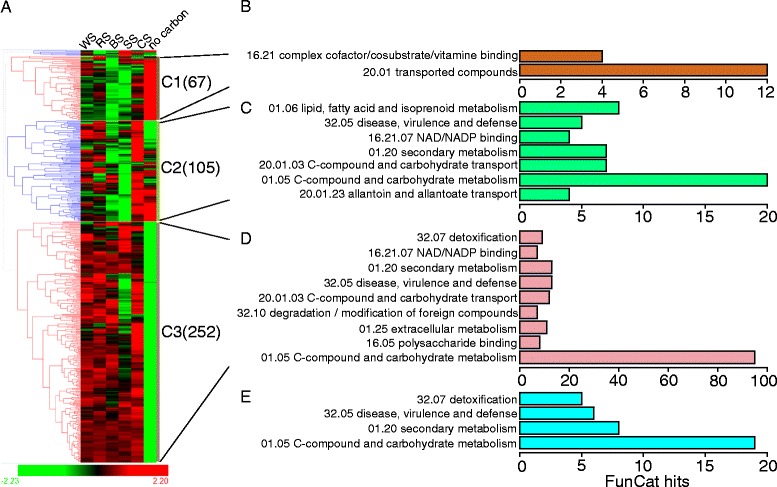
**Hierarchical clustering and functional category analysis of 430-gene biomass commonly induced core set (BICS).** BS, barley straw; C1, cluster 1; C2, cluster 2; C3, cluster 3; CS, corn straw; RS, rice straw; SS, soybean straw; WS, wheat straw. **(A)** Hierarchical clustering analysis of log_2_ fold changes in each crop residue versus the sucrose condition. The gene numbers for each cluster are shown in brackets. “No carbon” data were derived from a reference [20]. **(B–E)** Functional category analysis [29] of C1, C2, C3 (biomass regulon, BR), and biomass unique set (BUS, including 88 genes) from C3 compared with three polysaccharide regulons (see Figure 6A), respectively. Categories with FunCat hits more than three and *P* value < 0.05 are shown.

The 430 genes commonly induced by all five crop residues (the BICS genes) were primarily assigned into three clusters using hierarchical clustering with comparison to the no-carbon condition [30] (Figure 5A; Additional file 5: Table S4, sheet 1). Clusters 1 (C1) and 2 (C2) genes were mostly upregulated by carbon starvation. These two clusters were mainly related to nutrient metabolism and transport and enzyme cofactors (Figure 5B, C). This result might indicate reliance on cellular organic metabolism, transformation, and energy homeostasis in carbon starvation conditions. The biggest cluster (C3) harbored 252 genes that were specifically induced by biomass (Figure 5A). This 252-gene set was upregulated on all five crop residues compared with both sucrose (carbon catabolite repression) and no-carbon (starvation) conditions and was denoted as the “biomass regulon” (BR) [20,22]. FunCat results showed that C-compound or extracellular carbon metabolism and polysaccharide binding were prevalent in the BR group (Figure 5D). Two recently characterized, conserved cellulose degradation regulators *clr-1* (NCU07705) and *clr-2* (NCU08042) [20] were also included in this set (Additional file 6: Table S5, sheet 1).

**Figure 6:**
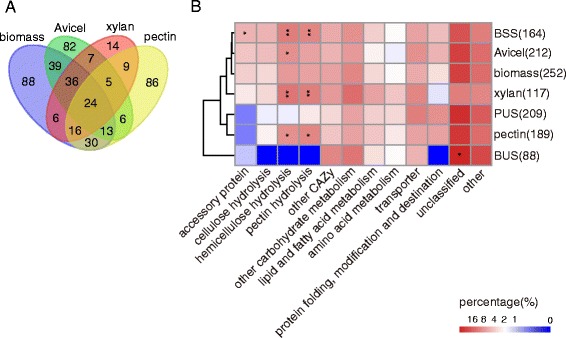
**Comparative analysis of 252 BR genes versus three polysaccharide regulons (Avicel, xylan, and pectin) in** ***Neurospora crassa*** **[** 20 **,** 22 **].** BSS, biomass shared set; BUS, biomass unique set; PUS, polysaccharide unique set. **(A)** Venn diagram of the BR compared with three polysaccharide regulons. **(B)** Functional enrichment of BR versus polysaccharide regulons. Gene numbers for each group are shown in brackets. The percentages of functional categories were displayed using a heat map. Enrichment analysis was performed by one-tailed Fisher’s exact test with the background of all lignocellulolytic regulon genes (BR plus three polysaccharide regulons with redundant genes removed). *P < 0.05; **P < 0.01.

The regulons of three main plant cell wall polysaccharides, cellulose (Avicel), hemicellulose (xylan), and pectin, have been published and include 212, 117, and 189 genes, respectively [20,22]. Venn diagram comparison of these three datasets showed that 164 BR genes were shared with the polysaccharide regulons, donated as the “biomass shared set” (BSS), but another 88 genes were not shared and comprised the “biomass unique set” (BUS) (Figure 6A). As shown in Figure 6A, the 252 BR genes observed in the present study, combined with the polysaccharide regulons identified previously (212 for Avicel [20], 117 for xylan [22], and 189 for pectin [22]), a total of 461 genes were obtained after removing the redundant ones. These 461 genes were then manually assigned to several functional categories based on FunCat [29] and CAZy [26,31] (Additional file 6: Table S5, sheet 2). Hierarchical clustering analysis of functional category showed that the BR was the closest to the Avicel regulon (Figure 6B). The 164-gene BSS was especially enriched for auxiliary activities family (mainly belonging to AA9, previously GH61), hemicellulase, and pectinase genes. The BUS consisted of some unclassified CAZy genes, carbon metabolism genes, and 38 other unclassified genes (Figure 5E; Additional file 6: Table S5, sheet 2). These genes might be of importance for plant biomass degradation and utilization [17]. For example, by checking the phenotypes of transcription regulator mutants from BUS, a limited characterized transcription factor *rca-1* (NCU01312) [32] was found to affect the expression and production of cellulase and xylanase (see mutant screening, below). Not surprisingly, the xylan and pectin regulons were enriched for hemicellulose and pectin hydrolytic genes but showed limited induction for AA genes (Figure 6B).

### The CAZome was induced on a large scale and functioned on various lignocellulosic materials as a conserved hydrolytic enzyme pool

**Figure 7:**
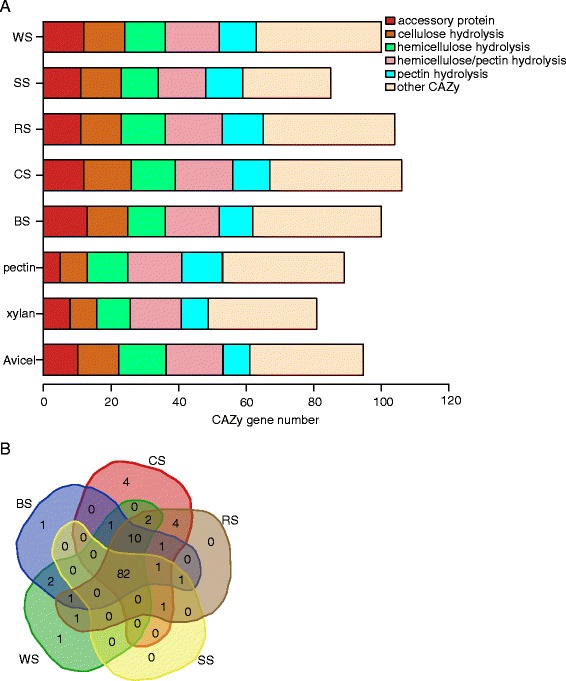
**Comparative analysis of** ***Neurospora crassa*** **CAZome induced by five crop residues and three polysaccharides (Avicel, xylan, and pectin)** **[** 20 **,** 22 **].** BS, barley straw; CS, corn straw; RS, rice straw; SS, soybean straw; WS, wheat straw. **(A)** Composition distribution of the CAZome of *N. crassa* induced by various lignocellulosic substrates. **(B)** Venn comparison of the CAZome induced by five crop residues.

Two hundred twenty-five genes in the *N. crassa* genome are predicted to encode glycosyl hydrolases based on the latest version of CAZy [31], called the CAZome (Additional file 7: Table S6, sheet 1). Of the 225 CAZy genes, approximately 100 were significantly upregulated threefold on most of the tested crop residues (Figure 7A and Additional file 7: Table S6, sheet 2). This upregulated gene set was slightly larger than those on any one of the three polysaccharides (Avicel, xylan, and pectin), especially xylan (Figure 7A). Moreover, the CAZy groups induced by the five crop residues displayed similar distributions of six CAZy functional categories; all five crop residues elicited an especially high number (11–13 of 14) of AA9 genes (Figure 7A). A Venn diagram of these five crop residue-induced CAZy groups showed that they shared the majority (82 genes) of their upregulated genes (Figure 7B).

### The transcriptional regulator *rca-1* is involved in lignocellulolytic enzyme synthesis for *N. crassa* grown on plant biomass

Multiple studies have already shown that several transcriptional regulators and a set of hydrolytic enzymes are tightly associated with polysaccharide degradation in *N. crassa* [20,21,33]. These genes were mainly identified under homo-polysaccharide conditions. This study showed that many genes were uniquely induced by various crop residues (Figure 6A). Screening the single gene deletion strain database from Fungal Genetics Stock Center (FGSC) [19] is an efficient way to identify critical components for plant cell wall degradation in *N. crassa* [20,22]. Thus, knockout strains of transcriptional regulators from the BUS (Figure 6A and Additional file 6: Table S5, sheet 2) were screened using Vogel’s minimal medium with 2% ground corn straw as the sole carbon source (“Methods”).

**Figure 8:**
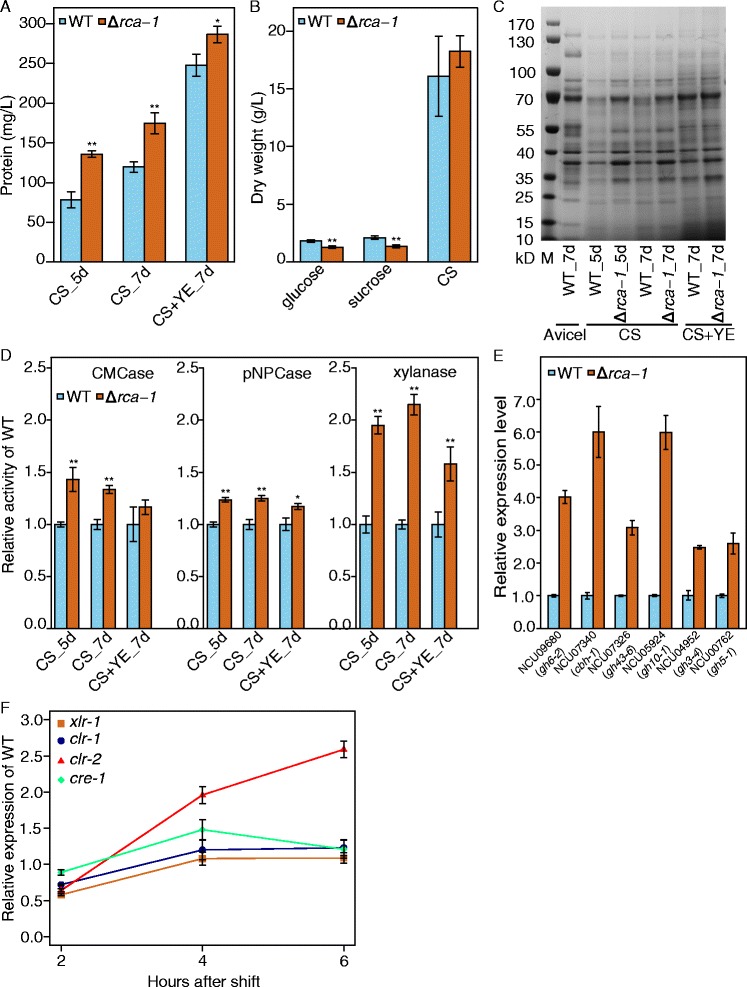
**Phenotype of mutant Δ** ***rca-1*** **versus wild type (WT) grown on corn straw.** CS, corn straw; M, protein marker; YE, yeast extract. **(A–D)** Secreted proteins, mycelial dry weight (mycelia grown on 2% glucose, or sucrose for 16 h, and 2% corn straw for 7 days), SDS-PAGE, and enzymatic activities, including cellulose hydrolysis activity (CMCase), exo-glucanase activity (pNPCase), and xylanase activity, of Δ*rca-1* versus WT when grown for the indicated times. **(E)** Induction of selected CAZy genes in WT and Δ*rca-1* mutant after transfer 16-h sucrose grown mycelia to corn straw for 4 h. **(F)** Time course of relative expression levels of plant cell wall-degrading associated regulators in Δ*rca-1* mutant versus WT after transfer from 16-h sucrose culture to corn straw for the indicated times. Values represent the means of at least three biological replicates; error bars show standard deviation. Statistical significance was performed using a two-tailed Student’s *t*-test. *P < 0.05; **P < 0.01.

A sporulation regulator (NCU01312) deletion mutant Δ*rca-1* secreted significantly more proteins than wild type (WT) when grown on corn straw (Figure 8A). The mycelia dry weight of Δ*rca-1* was slightly higher than that of WT, but not significantly so by Student’s *t*-test (Figure 8B). However, the Δ*rca-1* mutant showed deficient growth on glucose and on sucrose (Figure 8B). This deficient phenotype was also observed on Avicel but not on xylan and pectin (Additional file 8: Figure S2), suggesting that the higher enzyme secretion in Δ*rca-1* was related to the plant biomass and not pure cellulose induction. Protein sodium dodecyl sulfate polyacrylamide gel electrophoresis (SDS-PAGE) of Δ*rca-1* showed similar banding patterns but remarkably increased protein abundances as compared with WT grown on corn straw (Figure 8C). These bands were probably NCU07340 (CBH-1, ~70 kD)/NCU09680 (GH6-2, ~70 kD), NCU07190 (GH6-3, ~40 kD), and NCU07326 (GH43-6, ~37 kD) based on a comparison with the SDS-PAGE of the *N. crassa* secretome from a previous report [12]. The CMCase, pNPCase, and xylanase activities confirmed this observation (Figure 8D), especially that of xylanase, which was approximately twofold higher xylanase activity than that of WT. The transcript abundance of all selected main cellulases (NCU09680, NCU07340, NCU04952, and NCU00762) and xylanases (NCU07326 and NCU05924) in mutant Δ*rca-1* grown for 16 h on sucrose and then transferred to corn straw for 4 h caused at least a twofold higher induction than in WT (Figure 8E). This finding was in accord with the greater extracellular protein levels and enzyme activities in Δ*rca-1* (Figure 8A, D), which were also observed when *N. crassa* was grown on BS, RS, and WS as the sole carbon sources (Additional file 9: Figure S3). Although the culture supernatant of SS showed low secreted protein levels (data not shown), 4 h induction by SS when transferred from 16 h of pre-growth on sucrose showed that the transcript abundances of cellulase and xylanase were elevated in the *rca-1* mutant (Additional file 9: Figure S3C).

**Figure 9:**
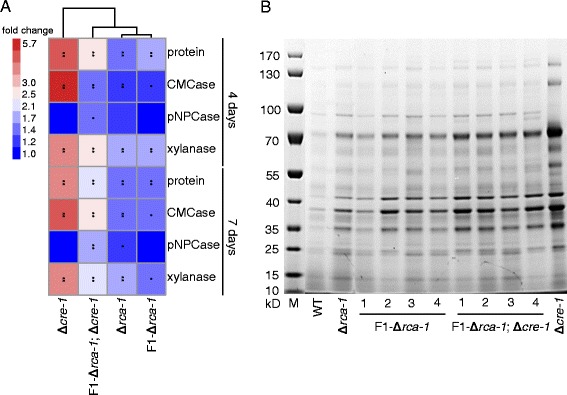
**Phenotype of Δ** ***rca-1*** × **Δ** ***cre-1*** **progeny.** F1-Δ*rca-1*, *rca-1* deletion progeny; F1-Δ*rca-1*; Δ*cre-1*, *rca-1* and *cre-1* double deletion progeny; M, protein marker. **(A)** Heat map of secreted proteins, cellulose hydrolysis activity (CMCase), exo-glucanase activity (pNPCase), and xylanase activity of progenies, Δ*rca-1*, and Δ*cre-1* grown on corn straw for 4 and 7 days compared with wild type (WT). Relative activities versus WT are displayed by color codes. Statistical significance was performed using a two-tailed Student’s *t*-test. *P < 0.05; **P < 0.01. **(B)** SDS-PAGE of secreted proteins of progenies, Δ*rca-1*, Δ*cre-1*, and WT when grown on corn straw for 4 days. Values represent the means of at least three biological replicates.

*N. crassa rca-1* is a homolog of the sporulation regulator *flbD* in *Aspergillus nidulans* [34]. It encodes a protein with a Myb-like DNA-binding domain that can complement the *A. nidulans flbD* mutant but has nearly unidentifiable effects on *N. crassa* conidiation [32]. However, loss of this gene significantly increased lignocellulolytic enzyme production in *N. crassa* when exposed to plant cell wall (Figure 8A, C and Additional file 9: Figure S3A). To further determine the regulatory differences in cellulase expression between Δ*rca-1* and WT, the transcript expression levels of major lignocellulase regulators in *N. crassa*, including *clr-1*/*2* (NCU07705/NCU08042) [20], *xlr-1* (NCU06971) [21], and *cre-1* (NCU08807) [33], were assessed by qRT-PCR. *clr-2* consistently displayed a two folds higher transcript level in Δ*rca-1* versus WT when mycelia pre-grown for 16 h in minimal medium with 2% sucrose were transferred to corn straw for 4–6 h (Figure 8F), indicating that *rca-1* may have a negative effect on *clr-2* expression when mycelia are exposed to plant straws. *clr-2* also showed remarkably higher expression level in the Δ*rca-1* mutant when mycelia were grown on soybean straw (Additional file 9: Figure S3C). Conversely, the transcript level of *rca-1* did not show any remarkable changes in Δ*clr-1*/*2*, Δ*xlr-1*, or Δ*cre-1* mutants versus WT [20,21,33]. All these observations suggest that *rca-1* affects the expression of plant polysaccharide-degrading enzyme genes by upstream regulation/modulation of the cellulose regulator *clr-2* when grown on plant biomass. Additionally, the double mutant F1-Δ*rca-1*; Δ*cre-1* (Additional file 10: Figure S4) had approximately two folds higher lignocellulolytic enzyme production than that of Δ*rca-1* (Figure 9) and significantly higher pNPCase activity than that of Δ*cre-1* (Figure 9A), suggesting a possible novel strategy of strain improvement for lignocellulase production by co-disruption of *rca-1* and *cre-1*.

## Discussion

Despite some differences in chemical composition and organization among five major crop residues, especially the dicotyledonous soybean straw, which was slightly more distinct than the other four monocot straws in composition as well as transcriptional response in *N. crassa*, transcriptome profiling showed a large conserved core response to these straws in the model cellulolytic fungus *N. crassa*, including a conserved CAZome. Of 430 core genes responding to growth on plant biomass, a 252-gene set was identified as the BR. Many genes (65%) in this set overlapped with either the Avicel, xylan, or pectin regulons, including cellulase genes, hemicellulase genes, pectinase genes, and LPMO genes (Additional file 7: Table S6, sheet 2), indicating that the polysaccharide block organization of plant straws extensively induced the *N. crassa* CAZome to degrade complex lignocellulosic substrates. Previous work [22] has identified a 29-gene set commonly induced by all three polysaccharides and suggested that this gene pool could be used for carbon scouting under any carbon-inducing condition. Twenty-four (82%) of these genes were also induced by crop straws in the present study (Figure 6A), supporting the hypothesis that a conserved gene set is involved in carbon scouting in *N. crassa*. A conserved response to crop straws increases the feasibility of universal plant biomass-based biorefinery by fungi, as in DMC. Further investigation of this core response during the plant biomass DMC process may offer potential targets for engineering strains to increase production of cell wall-degrading enzymes and the end-product conversion rate.

Eighty-eight genes were upregulated only on plant biomass, rather than on Avicel, xylan, or pectin [20,22]. Athough most genes in this set encoded unknown or unclassified proteins, they could be a resource for identifying novel components involved in lignocellulose deconstruction [17]. For example, the sporulation regulator *rca-1* (NCU01312) screened from this gene set dramatically increased lignocellulase production in *N. crassa* when grown on plant biomass but not on Avicel. Previous studies show that *rca-1* can complement *flbD* deletion in *A. nidulans*, but deletion had no detectable sporulation defect in *N. crassa* [32]. Intriguingly, loss of the *flbD* downstream conidiation regulator *brlA* in *Penicillium decumbens* not only blocked conidiation but also significantly improved cellulase production when grown on cellulose supplemented with wheat bran [35]. A bioinformatics search predicted that many sequences upstream of CAZy genes harbor *BrlA*-binding sites in *P. decumbens* [35], but evidence for a direct binding interaction has not yet been revealed. The RCA-1 regulator is a MYB family member that contains two imperfect repeats [32] for binding the *cis*-element 5′-AACTGNCW-3′ [36]. Searching the upstream 1-kb promoters of 225 CAZy genes by PATSER in RSAT [37] yielded 51 promoter regions containing candidate Myb-binding sites, but most harbored only one or two sites, with the exception of NCU07035 (Additional file 11: Table S7). The gene NCU07035, a member of glycosyl hydrolase family 18 (mainly representing chitinase), showed elevated expression levels when grown on all five tested crop residues (Additional file 3: Table S2) and also on Avicel when grown for 30 h [12]. Although the transcript of NCU07035 was not highly expressed, the role of its family in *N. crassa* grown on lignocellulose needs to be further investigated.

Examination of the expression levels of lignocellulolytic regulators *xlr-1*, *clr-1*/*2*, and *cre-1* showed that *clr-2* was upregulated in the *rca-1* deletion mutant compared with WT, indicating that the mutant might enhance the expression of biomass-degrading genes via de-repression of *clr-2*. Cellulolytic transcription factors *clr-1/2* were necessary to fully induce all major cellulase and some major hemicellulase genes in *N. crassa* [20]. Notably, induction of *clr-2* is dependent on *clr-1* in *N. crassa*. Therefore, RCA-1 might repress *clr-2* expression by directly regulating and (or) interacting with CLR-1 to modulate its induction, although *clr-1* was not suppressed by RCA-1 at the mRNA level (Figure 8F). Additionally, deletion of *rca-1* led to slower growth on glucose or sucrose than in the WT (Figure 8B) and also observed that *rca-1* was de-repressed when extracellular glucose levels increased (unpublished data), suggesting that this sporulation regulator plays a role in carbon or glucose metabolism/signaling. However, how this sporulation regulator in *N. crassa* affects cell growth in carbon-depleted or -plentiful environment requires further investigation of the correlation between colony survival and sporulation as well as biochemical identification of the targets of conidiation regulators in *N. crassa*.

Given that *rca-1* influences production of plant biomass-degrading enzymes on a wide spectrum of biomass substrates, *rca-1* might be an advantageous target for engineering the biorefinery of plant biomass through DMC. Functional characterization of BUS genes is a potential strategy to gain a more complete view of *N. crassa* growth on plant biomass. Further engineering of growth properties on plant biomass in conjunction with DMC product yield based on BUS might facilitate the fungal bioconversion process.

## Conclusions

Transcriptional profiling of *N. crassa* grown on different plant biomass revealed a conserved core gene set for plant cell wall utilization, including a conserved pool of CAZy genes. Eighty-eight genes (the biomass unique set, BUS) were specifically induced by plant biomass but not by Avicel, xylan, or pectin. Deletion of one of these genes, the sporulation transcription factor *rca-1*, was identified as beneficial for the production of lignocellulases on plant straw and might be a potential target to engineer strains for biomass-based biorefinery through direct microbial conversion technology.

## Methods

### Strains, media, and culture conditions

The *N. crassa* WT strain (FGSC 2489) and the *rca-1* deletion strain (NCU01312, FGSC 11209) were obtained from FGSC [38]. Mutant Δ*cre-1* was a gift from the laboratory of Professor N. Louise Glass. For RNA-Seq preparation, conidia of *N. crassa* WT were inoculated at 10^6^ conidia/mL into 100 mL 1× Vogel’s salts (minimal medium) with 2% (*w*/*v*) sucrose for 16 h or with 2% (*w*/*v*) of the indicated ground crop-straw carbon sources for 30 h and grown at 25°C with constant light shaking at 200 rpm. The 50× Vogel’s salts were prepared as previously described [39]. The straws included BS, CS, RS, SS, and WS were harvested from Anhui Province, China, and mechanically ground. Mycelia grown for 30 h on various straws were photographed under a microscope (OLYMPUS, Japan).

For protein assays, 2 mL of culture supernatants were collected at each time point, centrifuged at 15,294 × *g* for 8 min to remove mycelia and stored at 4°C for analysis within 1 day or at −20°C for SDS-PAGE. For media shift experiments, *N. crassa* cultures were first grown on 100 mL 1× Vogel’s salts supplemented with 2% (*w*/*v*) sucrose for 16 h, and mycelia were filtered through six layers of gauze and immediately washed with sterilized water at least five times. Then, mycelia were transferred into new media containing 100 mL 1× Vogel’s salts with 2% (*w*/*v*) ground straws for the indicated time (2, 4, and 6 h) at 25°C, 200 rpm.

For transcription factor mutant screening, conidia were inoculated at 10^6^ conidia/mL into a 250-mL flask with 100 mL 1× Vogel’s minimal medium with 2% ground corn straw as the sole carbon source then cultured for 7 days at 25°C, with shaking at 200 rpm under constant light.

### RNA extraction, sequencing, and data analysis

Cultured mycelia were harvested after the indicated growth time via filtration and immediately frozen in liquid nitrogen. Total RNA from frozen samples was isolated with TRIzol reagent (Invitrogen, Carlsbad, CA, USA) as previously described [12] and further treated with DNase I (RNeasy Mini Kit, QIAGEN, Hilden, Germany). RNA concentration was measured with a Nanodrop 2000c (Thermo Scientific, Waltham, MA, USA), and RNA integrity was checked by agarose gel electrophoresis. The total RNA of two biological replicate samples was extracted separately, mixed together after measuring the quality of each sample, and used for high-throughput RNA sequencing.

The qualified RNA was prepared using the standard protocol from Shenzhen BGI (Shenzhen, China) and sequenced on the Illumina HiSeq™ 2000 platform (San Diego, CA, USA). Prior to reads mapping, adaptors and low quality reads were removed based on the BGI standard process (www.genomics.cn). Total clean reads were mapped against predicted transcripts from the *N. crassa* OR74A genome (version 12) [40] using TopHat (version 2.0.8b) [41] with at most two mismatches. Raw counts of reads mapped to unique exons were calculated by HTSeq (version 0.6.0) [42] and used for both normalizing transcript abundance (RPKM, reads per kilobase per million mapped reads [43]) and differential gene expression analysis using GFOLD (version 1.1.0) [27] and DEGseq [28]. Genes with GFOLD ≥ 1.6 (log_2_ fold change ≥ 1.6) and DEGseq *P* value < 1e-4 were considered significantly differentially expressed between growth conditions. RNA-Seq raw data are available at the Gene Expression Omnibus under accession number GSE60986. The GFOLD values of genes commonly upregulated by the five crop residues were hierarchically clustered with HCE software (version 3.5) [30]. The complete linkage method with Euclidean distance as the similarity metric was used for cluster generation.

For PCA, the RNA-Seq reads of *N. crassa* grown on sucrose, BS, CS, RS, SS, and WS were the input data of DESeq [44], which calculated the eigenvectors for each sample by eigenvalue decomposition of the corresponding sample covariance matrix. The first two principle components of each eigenvector were plotted by it [44], with each point representing a biological sample. This type of analysis is useful for determining of sample-to-sample distances and batch effects [44]. Although PCA represents the total variance, the PCA plot displayed only the most important component of each sample.

### Quantitative real-time PCR

qRT-PCR was performed using the iScript cDNA Synthesis Kit and IQ SYBR Green Supermix (Bio-Rad, Hercules, CA, USA) or SYBR Green Realtime PCR Master Mix (TOYOBO, Osaka, Japan) according to the manufacturers’ instructions on a CFX96 real-time PCR detection system (Bio-Rad). Each reaction was done in triplicate. The actin gene (NCU04173) was used as an endogenous control for all experiments. All primers used in this study are listed in Additional file 12: Table S8. Relative expression level of each gene was calculated using the Livak method (2^−ΔΔCt^).

### Measurement of crop residue composition

Agricultural crop residue composition was measured according to the analytical procedure of the National Renewable Energy Laboratory (NREL; http://www.nrel.gov/). Briefly, 300 ± 1 mg biomass powder was transferred to a capped vessel with the addition of 3 mL 72% (*w*/*w*) sulfuric acid and incubated at 30°C in a water bath for 1 h. During this time, the vessel was vortexed at a maximum speed of 5 s every 10 min. After incubation, 84 mL deionized water was added, and the mixture was carefully transferred to a capped bottle. The mixture was then autoclaved for 1 h at 121°C. The hemicellulosic glucose analysis was previously described [45]. Briefly, 50 ± 1 mg biomass was shortly mixed with 14.5 mL of 4% (*w*/*w*) sulfuric acid before autoclaving. Finally, the concentration of monosaccharides was determined by high-performance liquid chromatography (HPLC; Waters e2695, Manchester, UK) using a Waters 2414 refractive index detector and Aminex HPX-87H Column (Bio-Rad). Elution was performed at 63°C with 5 mM sulfuric acid at a flow rate of 0.6 mL/min. The content of cellulose and hemicellulose in the crop residues was calculated by the following formulas: %cellulose (*w*/*w*) = [(*C*_total glucose_ − *C*_hemicellulosic glucose_) × 86.73 × 0.9 × 100]/300, %hemicellulose (*w*/*w*) = [(*C*_xylose_ + *C*_arabinose_) × 86.73 × 0.88 × 100 + *C*_hemicellulosic glucose_ × 86.73 × 0.9 × 100]/300, where, *C*_monosaccharide_ is the concentration (mg/mL) of a sugar as determined by HPLC.

For acid insoluble lignin, the precipitate of the autoclaved mixture above was dried at 75°C until constant weight then ashed using a muffle furnace with a ramping program at 575°C for 3 h. The sample was weighed again after cooling. The content of acid insoluble lignin was calculated by the formula: %acid insoluble lignin (*w*/*w*) = (*W*_precipitation_ − *W*_acid-insoluble ash_) × 100/300. The absorbance at 320 nm of the hydrolysis supernatant was measured with a UV-1800 spectrophotometer (Shimadzu, Kyoto, Japan) and used to calculate acid soluble lignin by the formula: %acid soluble lignin (*w*/*w*) = OD_320_ × 86.73 × 100/30/300.

For ash determination, 600 ± 1 mg dried biomass powder was ashed using a muffle furnace at 575°C for 3 h. The content was calculated by the formula: %ash (*w*/*w*) = *W*_ash_ × 100/600.

### Enzyme activity and dry weight assays

The total extracellular protein content was determined using the Bradford (Bio-Rad) method with BSA as a standard. The endo-glucanase and endo-xylanase activities were measured using the azo-CMC/xylan kit (Megazyme, Wicklow, Ireland) according to the manufacturer’s instructions. The exo-glucanase activity was assessed using *p*-nitrophenol-D-cellobioside (pNPC; Sigma-Aldrich) as the substrate. Briefly, a 250-μL culture supernatant that was diluted with 50 mM sodium citrate (pH 4.8) was added to 250 μL of 1 mg/mL pNPC, then incubated immediately for 10 min at 50°C. The reaction mixture was terminated by adding 500 μL of 1 M sodium carbonate and determined at 420 nm by a UV-1800 spectrophotometer (Shimadzu). One unit (U) of exo-glucanase activity was defined as the amount of enzyme that liberates 1 μmol of pNP per min.

Mycelia grown on sucrose or glucose for 16 h were harvested, dried, and weighed. Biomass dry weight of corn straw cultures was indirectly measured as previously described [46]. Briefly, a 5-mL culture broth was centrifuged at 3,220 × *g* for 5 min, the supernatant was discarded, and 3 mL acetic acid (80%, *v*/*v*):nitrate (10:1, *v*/*v*) reagent was added to solubilize fungal biomass by boiling in water for 2 h. The reaction mixture was then centrifuged, dried, and weighed. Mycelial dry weight was defined as the dry weight of the original 5-mL culture minus that of the reaction mixture.

### Statistical significance tests and data plotting

Statistical significance tests between two conditions were performed using a two-tailed Student’s *t*-test. Enrichment analysis involved the identification of one functional category or gene group that was overrepresented in a whole gene collection. Significant enrichment was analyzed by a one-tailed Fisher’s exact test (http://www.langsrud.com/stat/fisher.htm) unless otherwise indicated. For all tests, *P < 0.05, **P < 0.01. Data were plotted using the R program platform (http://www.r-project.org/). Venn diagrams were constructed with a web tool from Ghent University (http://bioinformatics.psb.ugent.be/webtools/Venn/).

## Additional files

Additional file 1: Table S1. Chemical composition of five crop residues. BS, barley straw; CS, corn straw; RS, rice straw; SS, soybean straw; WS, wheat straw. Values represent the mean ± standard deviation of at least three replicates. ^a^The percentages of three monosaccharides in each residual hemicellulose are shown in brackets in the order hemicellulosic glucose, xylose, and arabinose. Additional file 2: Figure S1. Phenotype of *Neurospora crassa* grown on five crop residues (2%, *w*/*v*) for 30 h and 2% sucrose for 16 h. Scale bar = 100 um. Additional file 3: Table S2. Gene expression profiles and differential expression analysis of RNA-Seq data. For all genes RPKM, log_2_ fold change, and *P* value on each crop residue versus sucrose were calculated. Additional file 4: Table S3. Functional category analysis (FunCat) of up/downregulated genes. Sheet 1: functional category analysis (FunCat) of upregulated genes excluding the 430-gene biomass commonly induced core set; sheet 2: functional category analysis (FunCat) of downregulated genes. Additional file 5: Table S4. Hierarchical clustering and functional category analysis of 430 biomass commonly induced core set genes. Sheet 1: clustering analysis of 430-gene biomass commonly induced core set (BICS); sheet 2: functional category analysis (FunCat) of 430-gene BICS. Additional file 6: Table S5. Functional classification of biomass and polysaccharide regulons. Sheet 1: regulons induced by various lignocellulosic substrates in *N. crassa*; sheet 2: functional classification of lignocellulolytic regulons. Additional file 7: Table S6. CAZome elicited by five crop residues. Sheet 1: the CAZome of *N. crassa* genome; sheet 2: upregulated CAZy genes of *N. crassa* induced by various lignocellulosic substrates. Additional file 8: Figure S2. Phenotype of mutant Δ*rca-1* versus wild type (WT) grown on three polysaccharides, Avicel for 7 days, xylan for 4 days, and pectin for 4 days. (A)–(D) Relative levels of secreted proteins, cellulose hydrolysis activity (CMCase), exo-glucanase activity (pNPCase), and xylanase activity of Δ*rca-1* versus WT grown on the indicated carbon source. Values represent the means of three replicates; error bars show standard deviation. Additional file 9: Figure S3. Phenotype of mutant Δ*rca-1* versus WT grown on four non-corn crop residues. (A) Relative levels of secreted proteins and xylanase activities of Δ*rca-1* versus WT grown on indicated crop straws for 4 days. (B) SDS-PAGE of secreted proteins of WT and Δ*rca-1*. (C) Induction of tested CAZy genes and lignocellulolytic regulators in WT and Δ*rca-1* mutant after transfer to soybean straw for 4 h from 16-h sucrose grown culture. Values represent the means of three replicates; error bars show standard deviation. Additional file 10: Figure S4. Genotypes of mutant Δ*rca-1* × Δ*cre-1* progeny identified by PCR and 1.5% (*w*/*v*) agarose gel electrophoresis. F1-Δ*rca-1*, the Δ*rca-1* progeny; F1-Δ*rca-1*; Δ*cre-1*, the *rca-1* and *cre-1* double deletion progeny; *hph*, hygromycin phosphotransferase gene; M, oligo-nucleotide marker. For each isolate, from left to right, PCR products present the genes *cre-1*, *hph* in *cre-1* locus, *rca-1*, and *hph* in *rca-1* locus. If one isolate had DNA bands only in the lanes “cre-1” and “rca-1”, it was wild type. Conversely, if one isolate had DNA bands in the lanes “cre-1-hph” and “rca-1-hph” and no bands in the “cre-1” or “rca-1” lanes, it was an F1-Δ*rca-1*; Δ*cre-1* progeny. If it only showed bands in “cre-1” and “rca-1-hph” lanes, it was an F1-Δ*rca-1* progeny. Additional file 11: Table S7. Myb-binding site prediction in the upstream 1-kb promoter region of the CAZome of *N. crassa*. “F” represents forward strand; “C” represents complement strand. Additional file 12: Table S8. Primers used in this study.

## Abbreviations

AA: Auxiliary activity family
BICS: Biomass commonly induced core set
BR: Biomass regulon
BS: Barley straw
BSS: Biomass shared set
BUS: Biomass unique set
CAZome: Carbohydrate-active enzyme proteome
CAZy: Carbohydrate-active enzyme
CE: Carbohydrate esterase
CMCase: Carboxy-methyl-cellulose enzyme
CS: Corn straw
DMC: Direct microbial conversion
FGSC: Fungal Genetics Stock Center
GEO: Gene expression omnibus
GH: Glycoside hydrolase
HPLC: High-performance liquid chromatography
LPMO: Lytic polysaccharide monooxygenase
PCA: Principle component analysis
pNPC: *p*-nitrophenol-D-cellobioside
pNPCase: *p*-nitrophenol-D-cellobioside enzyme
PUS: Polysaccharides unique set
qRT-PCR: Quantitative real-time reverse transcription polymerase chain reaction
RPKM: Reads per kilobase per million mapped reads
RS: Rice straw
SDS-PAGE: Sodium dodecyl sulfate polyacrylamide gel electrophoresis
SS: Soybean straw
WT: Wild type
YE: Yeast extract

## References

[1] Kubicek CP (2012). Fungi and lignocellulosic biomass.

[2] Carroll A, Somerville C (2009). Cellulosic biofuels. Annu Rev Plant Biol..

[3] Sarkar N, Ghosh SK, Bannerjee S, Aikat K (2012). Bioethanol production from agricultural wastes: an overview. Renew Energ..

[4] Menon V, Rao M (2012). Trends in bioconversion of lignocellulose: biofuels, platform chemicals & biorefinery concept. Prog Energ Combust..

[5] Popper ZA, Michel G, Hervé C, Domozych DS, Willats WGT, Tuohy MG (2011). Evolution and diversity of plant cell walls: from algae to flowering plants. Annu Rev Plant Biol..

[6] Alvira P, Tomás-Pejó E, Ballesteros M, Negro MJ (2010). Pretreatment technologies for an efficient bioethanol production process based on enzymatic hydrolysis: a review. Bioresour Technol..

[7] Parisutham V, Kim TH, Lee SK (2014). Feasibilities of consolidated bioprocessing microbes: from pretreatment to biofuel production. Bioresour Technol..

[8] Lynd LR, Zyl WH, McBride JE, Laser M (2005). Consolidated bioprocessing of cellulosic biomass: an update. Curr Opin Biotechnol..

[9] Roche CM, Glass NL, Blanch HW, Clark DS (2014). Engineering the filamentous fungus *Neurospora crassa* for lipid production from lignocellulosic biomass. Biotechnol Bioeng..

[10] Xu Q, Singh A, Himmel ME (2009). Perspectives and new directions for the production of bioethanol using consolidated bioprocessing of lignocellulose. Curr Opin Biotechnol..

[11] Kolbusz MA, Di Falco M, Ishmael N, Marqueteau S, Moisan M-C, Baptista CS (2014). Transcriptome and exoproteome analysis of utilization of plant-derived biomass by Myceliophthora thermophila. Fungal Genet Biol..

[12] Tian C, Beeson WT, Iavarone AT, Sun J, Marletta MA, Cate JHD (2009). Systems analysis of plant cell wall degradation by the model filamentous fungus *Neurospora crassa*. Proc Natl Acad Sci U S A..

[13] Delmas S, Pullan ST, Gaddipati S, Kokolski M, Malla S, Blythe MJ (2012). Uncovering the genome-wide transcriptional responses of the filamentous fungus *Aspergillus niger* to lignocellulose using RNA sequencing. PLoS Genet..

[14] Berka RM, Grigoriev IV, Otillar R, Salamov A, Grimwood J, Reid I (2011). Comparative genomic analysis of the thermophilic biomass-degrading fungi *Myceliophthora thermophila* and *Thielavia terrestris*. Nat Biotech..

[15] Bischof R, Fourtis L, Limbeck A, Gamauf C, Seiboth B, Kubicek CP (2013). Comparative analysis of the *Trichoderma reesei* transcriptome during growth on the cellulase inducing substrates wheat straw and lactose. Biotechnol Biofuels..

[16] Ries L, Pullan S, Delmas S, Malla S, Blythe M, Archer D (2013). Genome-wide transcriptional response of Trichoderma reesei to lignocellulose using RNA sequencing and comparison with *Aspergillus niger*. BMC Genomics..

[17] Znameroski EA, Glass NL (2013). Using a model filamentous fungus to unravel mechanisms of lignocellulose deconstruction. Biotechnol Biofuels..

[18] Colot HV (2006). A high-throughput gene knockout procedure for *Neurospora* reveals functions for multiple transcription factors. Proc Natl Acad Sci U S A..

[19] Dunlap JC, Borkovich KA, Henn MR, Turner GE, Sachs MS, Glass NL (2007). Enabling a community to dissect an organism: overview of the *Neurospora* functional genomics project. Adv Genet..

[20] Coradetti ST, Craig JP, Xiong Y, Shock T, Tian C, Glass NL (2012). Conserved and essential transcription factors for cellulase gene expression in ascomycete fungi. Proc Natl Acad Sci U S A..

[21] Sun J, Tian C, Diamond S, Glass NL (2012). Deciphering transcriptional regulatory mechanisms associated with hemicellulose degradation in *Neurospora crassa*. Eukaryot Cell..

[22] Benz JP, Chau BH, Zheng D, Bauer S, Glass NL, Somerville CR (2014). A comparative systems analysis of polysaccharide-elicited responses in *Neurospora crassa* reveals carbon source-specific cellular adaptations. Mol Microbiol..

[23] Glass NL, Schmoll M, Cate JH, Coradetti S (2013). Plant cell wall deconstruction by ascomycete fungi. Annu Rev Microbiol..

[24] Gang L, Lei S (2007). Quantitative appraisal of biomass energy and its geographical distribution in China. J Nat Resour..

[25] Kim S, Dale BE (2004). Global potential bioethanol production from wasted crops and crop residues. Biomass Bioenerg..

[26] Cantarel BL, Coutinho PM, Rancurel C, Bernard T, Lombard V, Henrissat B (2009). The Carbohydrate-Active EnZymes database (CAZy): an expert resource for glycogenomics. Nucleic Acids Res..

[27] Feng J, Meyer CA, Wang Q, Liu JS, Liu XS, Zhang Y (2012). GFOLD: a generalized fold change for ranking differentially expressed genes from RNA-seq data. Bioinformatics..

[28] Wang L, Feng Z, Wang X, Wang X, Zhang X (2010). DEGseq: an R package for identifying differentially expressed genes from RNA-seq data. Bioinformatics..

[29] Ruepp A, Zollner A, Maier D, Albermann K, Hani J, Mokrejs M (2004). The FunCat, a functional annotation scheme for systematic classification of proteins from whole genomes. Nucleic Acids Res..

[30] Seo J, Bakay M, Chen Y-W, Hilmer S, Shneiderman B, Hoffman EP (2004). Interactively optimizing signal-to-noise ratios in expression profiling: project-specific algorithm selection and detection p-value weighting in affymetrix microarrays. Bioinformatics..

[31] Levasseur A, Drula E, Lombard V, Coutinho PM, Henrissat B (2013). Expansion of the enzymatic repertoire of the CAZy database to integrate auxiliary redox enzymes. Biotechnol Biofuels..

[32] Shen W-C, Wieser J, Adams TH, Ebbole DJ (1998). The *Neurospora rca-1* gene complements an *aspergillus flbD* sporulation mutant but has no identifiable role in *Neurospora* sporulation. Genetics..

[33] Sun J, Glass NL (2011). Identification of the CRE-1 Cellulolytic regulon in Neurospora crassa. PLoS ONE..

[34] Wieser J, Adams TH. *flbD* encodes a Myb-like DNA-binding protein that coordinates initiation of *Aspergillus nidulans* conidiophore development. Genes Dev. 1995;9:491–502. 10.1101/gad.9.4.491 7883170

[35] Qin Y, Bao L, Gao M, Chen M, Lei Y, Liu G (2013). *Penicillium decumbens* BrlA extensively regulates secondary metabolism and functionally associates with the expression of cellulase genes. Appl Microbiol Biotechnol..

[36] Mathelier A, Zhao X, Zhang AW, Parcy F, Worsley-Hunt R, Arenillas DJ (2014). JASPAR 2014: an extensively expanded and updated open-access database of transcription factor binding profiles. Nucleic Acids Res..

[37] Thomas-Chollier M, Defrance M, Medina-Rivera A, Sand O, Herrmann C, Thieffry D (2011). RSAT 2011: regulatory sequence analysis tools. Nucleic Acids Res..

[38] McCluskey K (2003). The Fungal Genetics Stock Center: from molds to molecules. Adv Appl Microbiol..

[39] Vogel HJ (1956). A convenient growth medium for *Neurospora*. Microb Genet Bull..

[40] Galagan JE, Calvo SE, Borkovich KA, Selker EU, Read ND, Jaffe D (2003). The genome sequence of the filamentous fungus *Neurospora crassa*. Nature..

[41] Trapnell C, Roberts A, Goff L, Pertea G, Kim D, Kelley DR (2012). Differential gene and transcript expression analysis of RNA-seq experiments with TopHat and Cufflinks. Nat Protoc..

[42] Anders S, Pyl PT, Huber W (2015). HTSeq - a Python framework to work with high-throughput sequencing data. Bioinformatics..

[43] Mortazavi A, Williams BA, McCue K, Schaeffer L, Wold B (2008). Mapping and quantifying mammalian transcriptomes by RNA-Seq. Nat Methods..

[44] Anders S, Huber W (2010). Differential expression analysis for sequence count data. Genome Biol..

[45] Bauer S, Ibáñez AB (2014). Rapid determination of cellulose. Biotechnol Bioeng..

[46] Updegraff DM (1969). Semimicro determination of cellulose in biological materials. Anal Biochem..

