# Supplementary figures and images for: A transcriptomic analysis of Neurospora crassa using five major crop residues and the novel role of the sporulation regulator rca-1 in lignocellulase production
Source: Biotechnol Biofuels. 2015 Feb 12;8:21. doi: 10.1186/s13068-015-0208-0 (PMC4330645; doi:10.1186/s13068-015-0208-0)

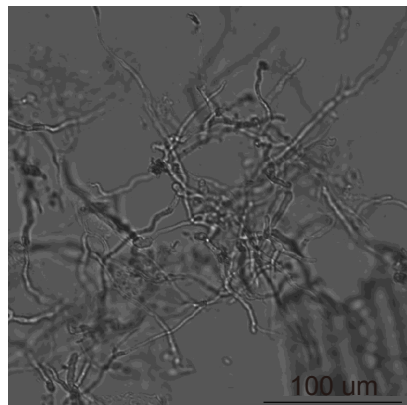

30 h on barley straw

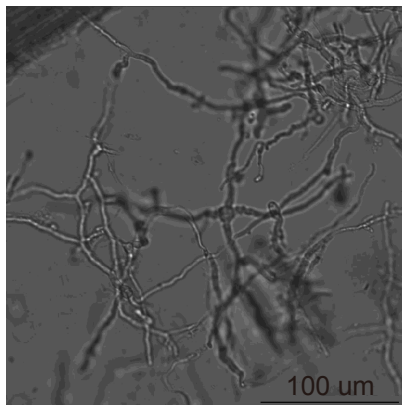

30 h on corn straw

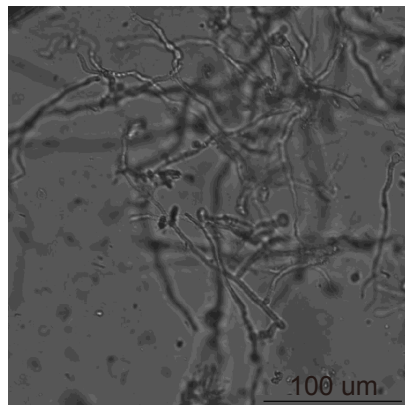

30 h on rice straw

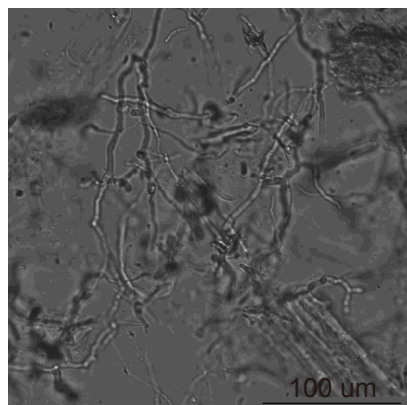

30 h on soybean straw

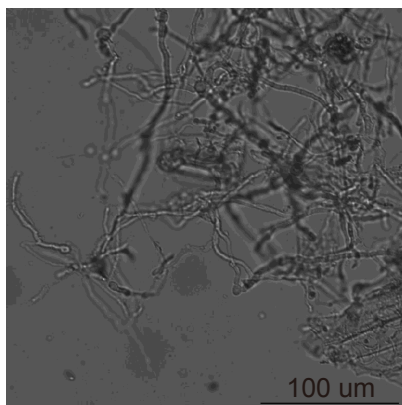

30 h on wheat straw

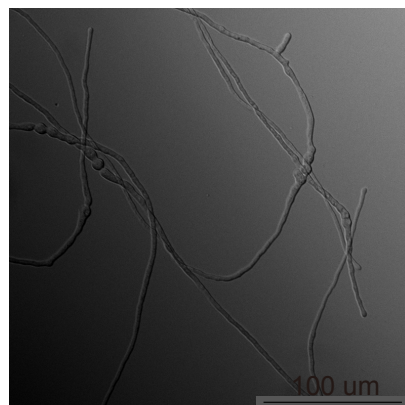

16 h on sucrose

Supplement: Additional file 2: Figure S1. — Phenotype of Neurospora crassa grown on five crop residues (2%, w/v) for 30 h and 2% sucrose for 16 h. Scale bar = 100 um. [file 13068_2015_208_MOESM2_ESM.pdf]

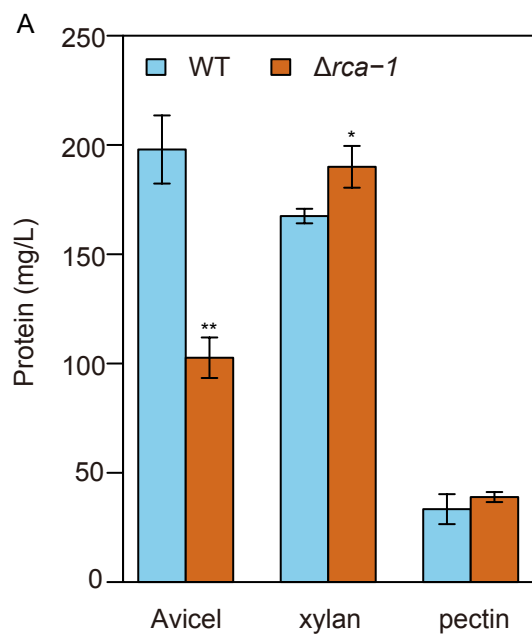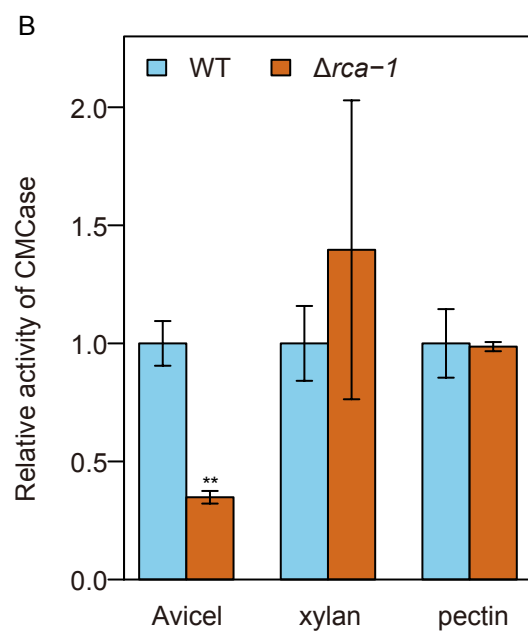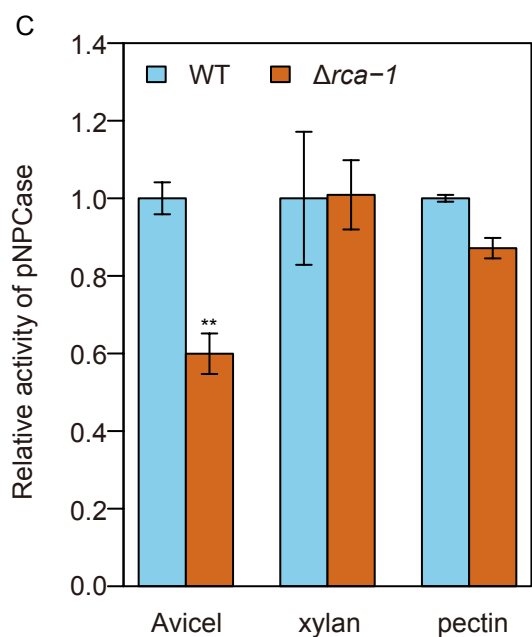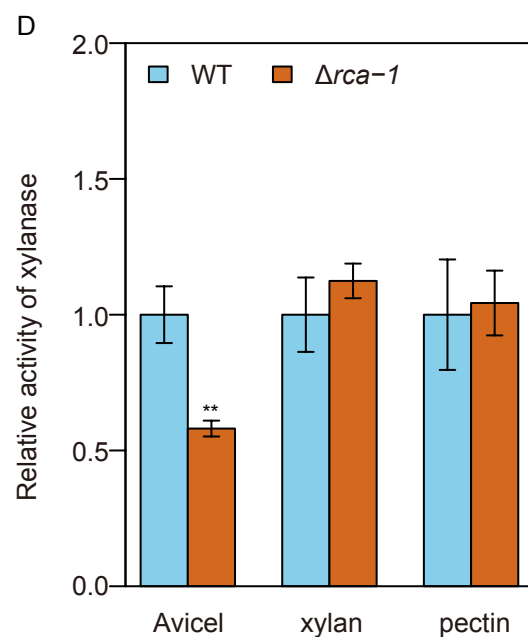

Supplement: Additional file 8: Figure S2. — Phenotype of mutant Δrca-1 versus wild type (WT) grown on three polysaccharides, Avicel for 7 days, xylan for 4 days, and pectin for 4 days. (A)–(D) Relative levels of secreted proteins, cellulose hydrolysis activity (CMCase), exo-glucanase activity (pNPCase), and xylanase activity of Δrca-1 versus WT grown on the indicated carbon source. Values represent the means of three replicates; error bars show standard deviation. [file 13068_2015_208_MOESM8_ESM.pdf]

A

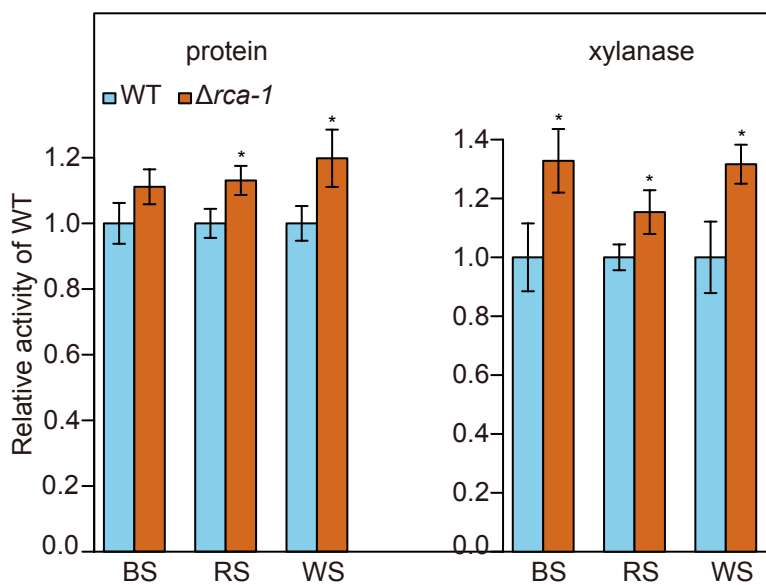

B

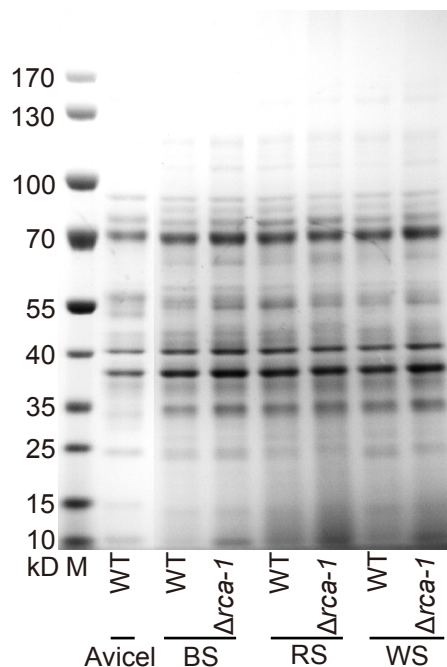

C

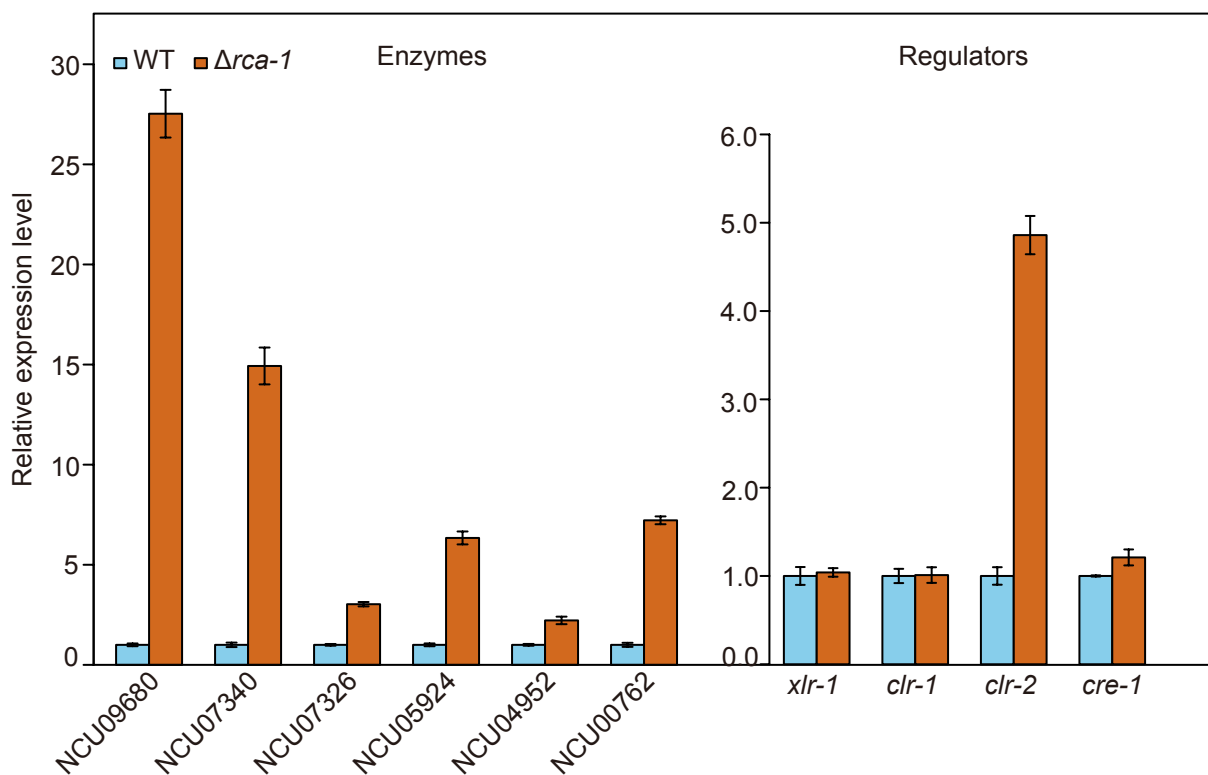

Supplement: Additional file 9: Figure S3. — Phenotype of mutant Δrca-1 versus WT grown on four non-corn crop residues. (A) Relative levels of secreted proteins and xylanase activities of Δrca-1 versus WT grown on indicated crop straws for 4 days. (B) SDS-PAGE of secreted proteins of WT and Δrca-1. (C) Induction of tested CAZy genes and lignocellulolytic regulators in WT and Δrca-1 mutant after transfer to soybean straw for 4 h from 16-h sucrose grown culture. Values represent the means of three replicates; error bars show standard deviation. [file 13068_2015_208_MOESM9_ESM.pdf]

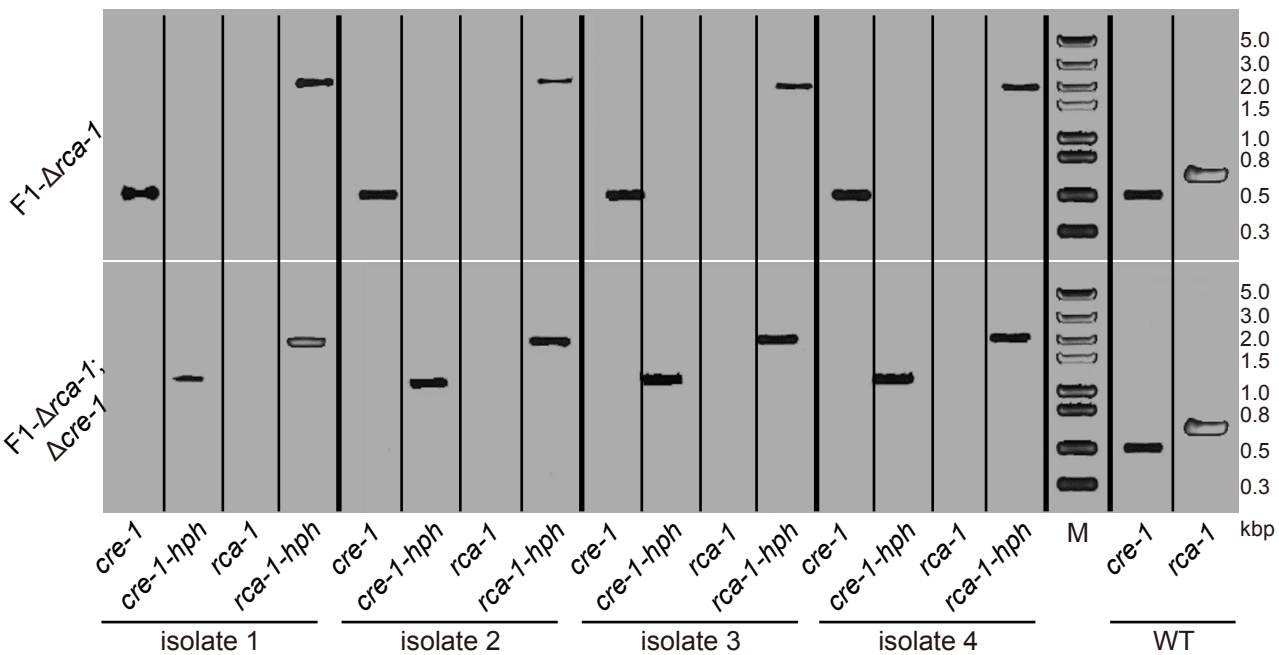

Supplement: Additional file 10: Figure S4. — Genotypes of mutant Δrca-1 × Δcre-1 progeny identified by PCR and 1.5% (w/v) agarose gel electrophoresis. F1-Δrca-1, the Δrca-1 progeny; F1-Δrca-1; Δcre-1, the rca-1 and cre-1 double deletion progeny; hph, hygromycin phosphotransferase gene; M, oligo-nucleotide marker. For each isolate, from left to right, PCR products present the genes cre-1, hph in cre-1 locus, rca-1, and hph in rca-1 locus. If one isolate had DNA bands only in the lanes “cre-1” and “rca-1”, it was wild type. Conversely, if one isolate had DNA bands in the lanes “cre-1-hph” and “rca-1-hph” and no bands in the “cre-1” or “rca-1” lanes, it was an F1-Δrca-1; Δcre-1 progeny. If it only showed bands in “cre-1” and “rca-1-hph” lanes, it was an F1-Δrca-1 progeny. [file 13068_2015_208_MOESM10_ESM.pdf]
